# Separated Channel Attention Convolutional Neural Network (SC-CNN-Attention) to Identify ADHD in Multi-Site Rs-fMRI Dataset

**DOI:** 10.3390/e22080893

**Published:** 2020-08-14

**Authors:** Tao Zhang, Cunbo Li, Peiyang Li, Yueheng Peng, Xiaodong Kang, Chenyang Jiang, Fali Li, Xuyang Zhu, Dezhong Yao, Bharat Biswal, Peng Xu

**Affiliations:** 1 School of Science, Xihua University, Chengdu 610039, China; zhangtao@mail.xhu.edu.cn; 2 School of Life Science and Technology, Center for Information in BioMedicine, University of Electronic Science and Technology of China, Chengdu 611731, China; cunboli@163.com (C.L.); yuehengp@umich.edu (Y.P.); 201821140226@std.uestc.edu.cn (C.J.); lfl_uestc@163.com (F.L.); xuyang508@163.com (X.Z.); dyao@uestc.edu.cn (D.Y.); 3 School of Bioinformatics, Chongqing University of Posts and Telecommunications, Chongqing 400065, China; pyli@cqupt.edu.cn; 4 Sichuan 81 Rehabilitation Centre, Chengdu University of TCM, Chengdu 611137, China; kxd1120@163.com

**Keywords:** deep learning, CNN, attention, ADHD

## Abstract

The accurate identification of an attention deficit hyperactivity disorder (ADHD) subject has remained a challenge for both neuroscience research and clinical diagnosis. Unfortunately, the traditional methods concerning the classification model and feature extraction usually depend on the single-channel model and static measurements (i.e., functional connectivity, FC) in the small, homogenous single-site dataset, which is limited and may cause the loss of intrinsic information in functional MRI (fMRI). In this study, we proposed a new two-stage network structure by combing a separated channel convolutional neural network (SC-CNN) with an attention-based network (SC-CNN-attention) to discriminate ADHD and healthy controls on a large-scale multi-site database (5 sites and *n* = 1019). To utilize both intrinsic temporal feature and the interactions of temporal dependent in whole-brain resting-state fMRI, in the first stage of our proposed network structure, a SC- CNN is used to learn the temporal feature of each brain region, and an attention network in the second stage is adopted to capture temporal dependent features among regions and extract fusion features. Using a “leave-one-site-out” cross-validation framework, our proposed method obtained a mean classification accuracy of 68.6% on five different sites, which is higher than those reported in previous studies. The classification results demonstrate that our proposed network is robust to data variants and is also replicated across sites. The combination of the SC-CNN with the attention network is powerful to capture the intrinsic fMRI information to discriminate ADHD across multi-site resting-state fMRI data.

## 1. Introduction

Attention Deficit Hyperactivity Disorder (ADHD) is one of the most common mental disorders among school-age children [1]. About 5% of children suffer from this disease [2], whose clinical symptoms are usually included distractibility, poor concentration, excessive activity, or weak self-control [3]. Theses ADHD behaviors have a negative effect on the course learning and normal life order of children. Thus, there is an urgent need for quantifiable and objective tools that may aid in early recognition of ADHD. However, at present, the clinical diagnosis of ADHD is mainly based on the behavior symptoms [4,5] that match with the Diagnostic and Statistical Manual of Mental Disorders (DSM) criteria, which is, unfortunately, subjective. The identification of neuroimaging biomarkers (functional features) and the application of an effective classification model are two important goals to discriminate ADHD from healthy controls (HCs).

Functional magnetic resonance imaging (fMRI) and machine learning as two powerful tools have been widely used to explore neural pathways and brain changes that occur in ADHD [6,7], which has the great capacity of extracting replicable brain functional features to classify the ADHD and HCs [8,9,10,11]. Especially, the resting-state fMRI has provided several significant information features, such as regional features (e.g., functional connectivity (FC), etc.) and global features (e.g., network properties, etc.), for ADHD diagnosis and classification. For example, Zhu et al. [12] trained a fisher-discriminant-analysis (FDA) classifier using the features of regional homogeneity (ReHo) based on resting-state fMRI to discriminate 20 subjects as ADHD or healthy and obtained an 85% leave-one-out cross-validation classification accuracy. Bernhardt et al. [13] used an overall LEFMSF algorithm to fuse structural texture features and FC and achieved an accuracy of 67% on the ADHD-200 data with the support vector machines (SVM) classifier. Tang et al. [14] applied a multi-affinity subspace clustering approach to FC feature for identifying ADHD and obtained the best performance of 96.2% for the single-site New York University Medical Center (NYU) dataset. 

These above-mentioned ADHD classifications are mainly based on the hand-crafted functional features derived from the precomputed measurement of brain architecture, although the relatively high accuracies are reported. The hand-crafted features (e.g., FC and Reho) are estimated mainly from the temporal and spatial correlations of blood–oxygen level-dependent (BOLD) signals, which are single and static measurements but leave the intrinsic information of the BOLD signal behind. The nature of the neural activity is highly dynamic even at rest [15,16], and the preprocessed BOLD time-series signals are believed to carry more useful information for discrimination. Meanwhile, although relatively high accuracy could be acquired for single-site data when applied to other site datasets, the trained model usually cannot achieve an acceptable performance [17,18,19] since the heterogeneity and variability exist in multi-site datasets [20,21], such as scan parameters and equipment, magnetic field strength, imaging length, age distribution, sample size, and male-to-female ratio. Indeed, it is a challenge to reliably identify the populations with brain disorders across larger and more heterogeneous datasets [20,21], and it is necessary to explore a reliable approach to simultaneously learn the discriminative features to achieve the multi-site classification of ADHD [22].

A convolutional neural network (CNN) has been applied to identify ADHD in multi-site fMRI data [23]. For instance, Riaz et al. [24] proposed a CNN-based deep learning structure, FCNet, to extract FC features from raw resting-state fMRI signals, and they found that the FCNet has superior discriminative power on the ADHD-200 dataset (the highest classification of 62.7% on the Peking dataset). Thereafter, Riaz et al. [25] further proposed an end-to-end deep model to learn FC features automatically and used a classification network to identify ADHD, which achieved an average accuracy of 67.9% by using three different ADHD-200 datasets. Zou [26] developed a three-dimensional CNN (3D-CNN) model to learn the local spatial patterns of ADHD from multi-model MRI features, and their proposed single modality 3D CNN architecture with the fractional amplitude of low-frequency fluctuations (fALFF) feature also achieved a mean accuracy of 66.04% on the ADHD-200 dataset.

However, those reported networks mainly applied a generalized network that considers the whole brain equally while neglecting the physiological basis that the different brain areas may provide the different information for the classification, i.e., the different brain area needs to be differently emphasized to extract the corresponding information. In this study, we proposed a separated channel attention convolutional network (SC-CNN-attention) to encode the time-series of the region of interest (ROI) directly for identifying the ADHD on multi-site rs-fMRI data. SC-CNN-attention is an end-to-end trainable network that can classify the concatenated features developed by a set of encoders. Our proposed method includes two network structures: (1) using a separated channel CNN that could handle long time-series data with different time points to learn the temporal feature in overall time-series signal for each brain region; and (2) and using attention network to capture temporal dependent features among regions. We extracted the 116 time-series signals using the automated anatomical labeling (AAL) template. The leave-one-site-out cross-validation strategy is used for inter-site classification, which is much closer to the practical clinical conditions. The goal of this study is to apply a new deep learning model to identify ADHD from a large multi-site resting-state fMRI dataset based on the original BOLD time-series signals.

## 2. Materials and Methods

### 2.1. Dataset

A publicly available dataset, the ADHD-200 data (*n* = 1019), was used for this study. The dataset comes from five different sites, including KKI, NI, NYU, OHSU, and Peking. The preprocessed ADHD dataset was downloaded from [27]. Table 1 showed the detailed information about the subjects [28].

### 2.2. Rs-fMRI Data Preprocessing

All datasets had been preprocessed by the neuroimaging analysis kit (NIAK) team using their preferred tools [28]. The detailed steps included: (1) slice timing correction; (2) motion correction; (3) quality control; (4) spatial normalization; (5) coregistration; (6) concatenation; (7) extraction of mask; (8) quality control for 4 and 5; (9) high-pass filtering; (10) correction of physiological noise; (11) resampling; and (12) spatial smoothing with 6-mm half-width Gaussian kernel. Concerning more parameters, please refer to [29].

### 2.3. Extraction of Time-Series

We first extracted the whole-brain resting-state fMRI BOLD time-series of all voxels within the same region based on the AAL-116 template [30], resulting in 116 time-series signals. Each region represented one single local signal channel. Then, the averaged the time-series signal across voxels in each region served as inputs to the separated single channel CNN network.

### 2.4. Separated Channel Convolutional Neural Network with an Attention Network (SC-CNN-Attention)

**Figure 1:**
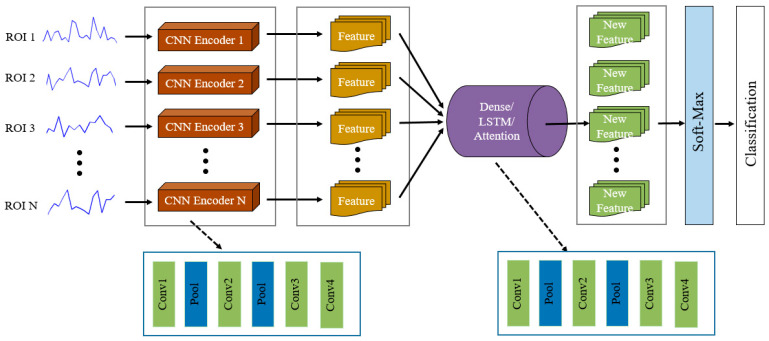
Architecture of the proposed SC-CNN-XX model for diagnosing attention deficit hyperactivity disorder (ADHD).

In our previous work [31], we proposed an end-to-end SC-CNN network framework to realize the training free motor imagery (MI) BCI system, where our model obtained a relatively high classification accuracy based on open MI EEG data. Motivated by this work, we proposed a new deep learning network model, SC-CNN-attention, to discriminate ADHD and HCs on large-scale multi-site fMRI data. The proposed framework consists of a two-stage network structure, with the first SC-CNN network to encode the fMRI time-series signal feature for each brain area (channel signal) and the second attention network to capture temporal interaction features among regions and extract fusion features. Figure 1 showed the network framework of the SC-CNN-XX model. To evaluate the generalization power of the attention layer (SC-CNN-Attention), we also concatenate SC-CNN with a fully connected layer (SC-CNN-Dense) and a bidirectional LSTM layer (SC-CNN-LSTM), to extract and fuse feature.

#### 2.4.1. SC-CNN Network

Most of the deep learning networks share a common CNN network to encode all input information; meanwhile, we did not consider the contribution of the different input signal to the feature learning. Here, our proposed model designed a separated CNN network for each ROI to learn abstract fMRI features, which could effectively capture the specificity of different fMRI time-series signals. All SC-CNNs had the same network structure and parameters. Such parameter sharing reduced the computational complexity of the model and inhibited the overfitting of the model. The parameter update of the encoders depended on the error of the ultimate classification result. During the training stage, each encoder adaptively adjusted the channel’s importance according to the gradient of a residual function, which reflected the levels of brain activities in different regions.

The length of resting-state fMRI time series was different among ADHD sites, to effectively handle the variance of the multi-site data, we averaged the feature maps extracted by each convolution filter at the output of the feature extractor so that each ROI or channel outputted a consistent feature that had the same length.

Besides, since the sample of ADHD patients was smaller than that of healthy people, which might result in a biased training process and make the output of the model more inclined to one category. In our present study, we used a special data generator to randomly select balance samples for training, which ensured the stability of the training and increased the credibility of the network output.

#### 2.4.2. Attention-Based Network

The attention-based network recently was widely applied to deep learning [32,33]. Attention gives the model the ability to distinguish the focus that should be focused on the numerous information, providing an important away to capture more reliable features [34]. In the first-stage network, the SC-CNN encoders obtained different channel-wise features, which were then concatenated as the input of the attention network. The attention network could adaptively learn the feature weights and give larger weight to more important features.

The attention mechanism is evaluated as:

(1) $$g_{n,n^{\prime }}=\tanh(W_{g}h_{n}+W_{g^{\prime }}h_{n^{\prime }}+b_{g})$$

(2) $$\alpha_{t,t^{\prime }}=\sigma (W_{a}g_{n,n^{\prime }}+b_{a})$$

where σ is the sigmoid function, which can be regarded as a threshold. The $W_{g}$ and $W_{g^{\prime }}$ is the corresponding weight matrix. The $W_{a}$ is a weight matrix of the nonlinear combination of $h_{n}$ and $h_{n^{\prime }}$. $b_{a}$ and $b_{g}$ are the offset vectors. Based on these parameters, we can get the hidden vector of $l_{n}$

(3) $$l_{n}=\sum_{n^{\prime }=1}^{N}\alpha_{t,t^{\prime }}\cdot h_{n^{\prime }}$$

where $l_{n}$ indicates the characteristic representation of the current brain region under the influence of other brain regions.

Several attention models have been introduced into deep learning, such as the soft attention model, the hard attention model, the global attention model, the local attention model, and the self-attention model [35,36]. In the current work, a novel soft attention model, called additive attention model, was selected and modified to extract weighted features.

#### 2.4.3. Classification Network

The integration of local features from multiple brain regions resulted in a series of novel features, which not only represented the information from the corresponding brain region but also contained the weighted information from other regions. These weighted features represented the contribution of the tasks. Subsequently, these weighted features would be sent into a fully connected layer with the soft-max activation function to predict the sample class.

### 2.5. Model Optimization

In the current study, the adaptive moment estimation (Adam) optimizer with an adaptive learning rate was utilized to minimize the cross-entropy between the real and predicted tags. The parameters in Adam were set by default [37]. Before the training stage, the Xavier initializer [38] was used to initialize the trainable weights randomly. The learning rate of the network is set as 0.01. Besides, to avoid the influence of over-fitting, the L2 regularization was used to restrict the complexity of model parameters. Specifically, we set the size of each small batch to 32 and randomly selected 16 samples from all ADHD sets and healthy sample sets to overcome the issue of unbalanced samples.

### 2.6. Leave-One-Site-Out

**Figure 2:**
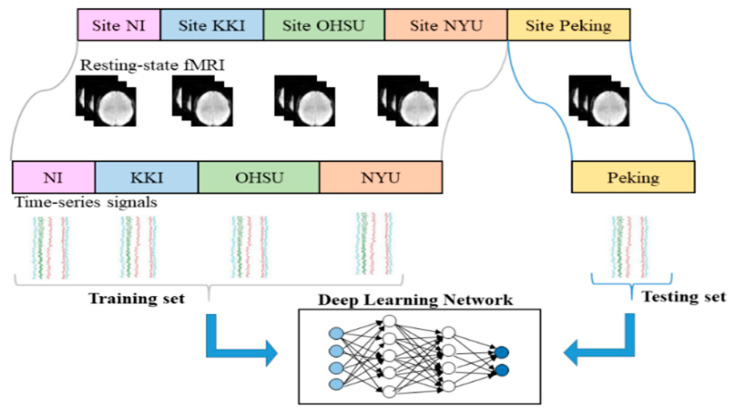
The leave-one-site-out cross-validation scheme.

To measure the classification accuracy of the model, the “leave-one-site-out” cross-validation was used. Assuming there were N datasets from N sites, we used N-1 sets as the training set to construct a network model and to learn the network parameters, and the data of the remaining site was used as the test set. With a circular pattern, each single site data was used as a test set to evaluate the performance of the model separately. The framework for multi-site ADHD date classification was shown in Figure 2.

## 3. Results

The multi-site ADHD classification experiments were performed on five different datasets. The SC-CNN-attention networks run in Ubuntu on a Core i7 PC with 40 GB RAM. Our proposed model was trained on an NVIDIA GTX 1070 GPU and implemented by Keras.

### 3.1. Classification Results Based on Multi-Site Data

**Figure 3:**
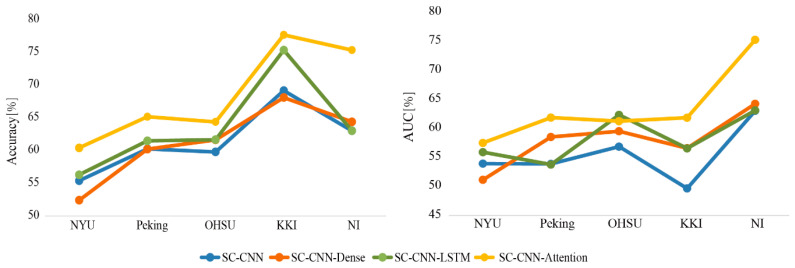
The accuracies and areas under the curves (AUCs) for each test site based on “SC-CNN+XX” models.

The accuracy and area under the curve (AUC) were used as the indexes to evaluate the performance of different deep learning network models. For comparison, SC-CNN, SC-CNN-Dense, and SC-CNN-LSTM models were also considered. As illustrated in Figure 3, the SC-CNN-attention deep network model achieved both higher accuracies and AUCs than the other methods on multi-site data by using the preprocessed time-series signals to learn features. We also found that the performance of the model increased along with the number of the training sample.

### 3.2. Overall Classification Results and Comparison

The classification results with the leave-one-site-out cross-validation structure validated the efficiency of our proposed SC-CNN-attention networks on multi-site fMRI datasets. Subsequently, we further calculated the overall accuracy for all sites and compared our method with several state-of-the-art methods. As listed in Table 2, our method outperformed the ADHD-200 competition teams [39], as well as the previous state-of-the-art deep learning model including FCNet [24], 3D-CNN [26], and DeepFMRI [25], achieving a 68.6% classification accuracy.

## 4. Discussion

In this study, we proposed a novel deep learning framework, SC-CNN-attention, to classify ADHD and HCs on large and multi-sites’ resting-state fMRI data, based solely on the preprocessed whole-brain time-series signals. The SC-CNN-attention architecture consists of two key components: (1) a separated channel CNN to learn different channel-wise features; (2) an attention-based network to adaptively learn channel-wise feature weights and to give more weight to important features. The results demonstrated that the SC-CNN-attention structure could successfully handle the multi-site dataset and obtain relatively high classification accuracy.

In general, aggregated heterogeneity datasets bring great challenges to develop suitable classifiers for psychiatric illnesses [17]. Our current work used five different sites’ dataset (*n* = 1019) that were larger than previous studies [25]. These datasets had no prior coordination, which was a real clinical application setting. Table 1 showed the differences between the datasets, especially in sample size, sex ratio, and time-series length. Our proposed SC-CNN-attention model did overcome the effect of the dataset heterogeneity. For each testing site, the classification accuracy and AUC indexes of our proposed model were higher than the other three models (i.e., SC-CNN, SC-CNN-dense, and SC-CNN-LSTM). In a recent autism spectrum disorder multi-site classification study, Abraham [19] found the performance of the model increased along with the number of the training sample. In our study, similar results were found on different sample sizes at different sites. For example, among the five testing sites, the NYU site had the largest sample size including 262 subjects whose data size was unfortunately imbalanced between ADHD and HCs, the classification results in the NYU site seemed not very high for all models whose highest accuracy was 60.4% (Figure 3). These results may imply that heterogeneity of the data causes false negative errors. Furthermore, we calculated the average accuracy to evaluate the performance of the model. The highest mean leave-one-site-out accuracy achieved 68.6% by using the SC-CNN-attention model (Table 2), which surpassed previous state-of-the-art deep learning methods [24,25,40] using the large and multi-site sample (less five sites).

Overall, our classification results indicated that the two-stage network structure had a better implementation effect. This might be attributed to three reasons. The first reason was that we directly inputted the preprocessed time-series signals into the model to learn the discriminative features. Unlike the traditional hand-crafted features [12,14,41], the resting-state BOLD signal reflected the special activated patterns of the brain [42], which carried more information. Furthermore, the hand-crafted features mainly represented the single and static brain activity measures, while the resting-state time-series signals measured dynamic brain activity [16,43]. Thus, the preprocessed time-series signals are facilitated to mine the significant features for ADHD classification using a deep learning method [18]. The second reason was that we proposed a novel SC-CNN model as the first-stage network. This module separately encoded the preprocessed time-series signal for each channel or each ROI and then generated channel-wise features. Such a network structure can not only reduce the complexity of the model but also retained all the channel’s learned features. Thus, the SC-CNN network could effectively capture the specificity of the different fMRI time-series signals, which facilitated the ADHD classification. The third one was that we combined the SC-CNN with the channel attention network. Recently, the attention mechanism has been widely used in deep learning tasks, such as natural language processing [36,44], image classification [33], object recognition [45], and speech recognition [32]. In essence, the attention model focuses on the key information linked to the context to optimize feature-representation [44]. In the current model, it returned a weighted mean of the input time-series signal, which was selected according to the relevance of each input signal. The attention mechanism highlighted the contribution of different brain regions to the classification of ADHD and took the interactions among brain regions into consideration, which was consistent with the neural mechanism of brain cognitive processes. Compared with the fully connected layer (dense) and LSTM layer, the attention mechanism layer achieved a relatively higher classification accuracy. This may be due to the fact that the fully connected network uses only one weight matrix for feature reconstruction, and its nonlinear ability is not strong. The attention mechanism in the deep learning model can learn the specificity and interaction pattern of time-series among different brain regions which have a great influence on the classification results.

Though our proposed deep learning method demonstrated good performance on multi-site data, it was still a challenge to replicate the findings across larger and more heterogeneous datasets and to generalize them to the real clinical situation. Indeed, in the current study, we only focused on the ADHD-200 dataset from five sites which included 1019 subjects. In future work, it would be essential to verify our deep network with more sample numbers and other aggregate samples (e.g., ABIDE). Another limitation was that the feature derived from the attention-based network might be too abstract to satisfy a biomarker standard; new metrics are thus necessary to not only capture the contribution of different brain regions for a classification task but increase the interpretability of the model and promote clinical applications in the future.

## 5. Conclusions

In this study, we presented an attention-based separated channel convolutional neural network for identifying ADHD and HCs on large multi-site resting-state fMRI datasets. It is a novel deep learning architecture, which directly inputs the original preprocessed BOLD time-series signals into the separated CNN model for each ROI to learn abstract features and weighs them by their contributions to the classification with the attention-based network. The results showed that the SC-CCN-attention model could handle the sizeable multi-site dataset and achieve a reliable and remarkable performance compared with the current state-of-the-art studies. These may help to better the diagnosis of ADHD and understand the neural mechanism of ADHD.

## Figures and Tables

**Table 1:** The demographic information for different sites.

Site	ADHD			HC			Volumes
	Age	Count	Total	Age	Count	Total	
KKI	8–13	10/15(F/M)	35	8–13	28/41(F/M)	69	152/119
NI	11–21	5/31(F/M)	36	12–26	25/12(F/M)	37	257
NYU	7–18	34/117(F/M)	151	7–18	55/56(F/M)	111	176/172
OHSU	7–12	13/30(F/M)	43	7–12	40/30(F/M)	70	78/50/73
Peking	8–17	10/92(F/M)	102	8–15	59/84(F/M)	143	236/231
Total	-	-	422	-	-	597	-

**Table 2:** Comparison of our proposed models with the average results of ADHD-200 competition teams, FCNet, 3D-CNN, and DeepFMRI in multi-site data.

	NYU	Peking	OHSU	KKI	NI	Overall Accuracy
Previous methods						
ADHD-200 competition [39]	35.2%	51.1%	65.4%	61.9%	57.0%	54.1%
FCNet [24]	58.5%	62.7%	-	-	60.0%	60.4%
3D-CNN [26]	-	62.9%	-	72.8%	-	67.8%
DeepFMRI [25]	73.1%	62.7%	-	-	67.9%	67.9%
Our models						
SC-CNN-Dense	55.4%	60.3%	59.8%	69.2%	63.0%	61.3%
SC-CNN	52.4%	60.2%	61.6%	68.1%	64.4%	61.5%
SC-CNN-LSTM	56.3%	61.5%	61.7%	75.3%	63.0%	63.6%
SC-CNN-Attention	***60.4%***	***65.2%***	***64.4%***	***77.7%***	***75.3%***	***68.6%***
